# Health-related quality of life and tobacco and alcohol consumption in Leber hereditary optic neuropathy in Sweden

**DOI:** 10.3389/fopht.2026.1791334

**Published:** 2026-06-15

**Authors:** Johan Hedström, Maria Nilsson, Martin Engvall, Pete A. Williams, Abinaya Priya Venkataraman

**Affiliations:** 1 Unit of Optometry, Division of Eye and Vision, Department of Clinical Neuroscience, Karolinska Institutet, Stockholm, Sweden; 2 Department of Molecular Medicine and Surgery, Karolinska Institutet, Stockholm, Sweden; 3 Division of Eye and Vision, Department of Clinical Neuroscience, St. Erik Eye Hospital, Karolinska Institutet, Stockholm, Sweden; 4 Centre for Eye Research Australia, Royal Victorian Eye and Ear Hospital, Melbourne, VIC, Australia

**Keywords:** alcohol, carriers, health-related quality of life, Leber hereditary optic neuropathy, LHON, smoking, snus

## Abstract

**Purpose:**

To investigate health-related quality of life (HRQoL), tobacco and alcohol consumption in Leber hereditary optic neuropathy (LHON) affected and carriers in Sweden.

**Methods:**

This cross-sectional case-control study included LHON affected and carriers from the Swedish LHON research registry. Age and sex matched healthy control (HC) references and references with other eye diseases were also included. Validated questionnaires on HRQoL, alcohol, smoking and smokeless-tobacco (snus) consumption were administered. The differences between groups and sub-groups (stratified for sex) were analyzed with ANOVA and independent samples t-test. Differences in prevalence of tobacco and alcohol consumption were evaluated with chi-square test.

**Results:**

32 LHON affected and 32 carriers were included. Mean age at inclusion were 49.1 ± 19.7 years and 48.2 ± 17.7 years respectively. LHON affected and carriers did not show any significant difference in HRQoL compared to HC reference group. However, the affected males had significantly lower scores for role limitation due to physical health than affected females (67.1 ± 36.4 vs. 95.0 ± 15.8, p = 0.019). A higher prevalence of smoking (20%) and snus-use (33%) was seen among LHON affected. One-fourth of LHON affected had harmful or hazardous alcohol consumption risk classification.

**Conclusions:**

The HRQoL was similar among the Swedish LHON cohort compared to HC references. Swedish LHON cohort showed a higher prevalence of smoking. Snus usage, and harmful or hazardous alcohol consumption were higher among LHON affected individuals.

## 1 Introduction

Quality of life (QoL) is well known to be affected in visual impaired subjects (1, 2). Leber hereditary optic neuropathy (LHON) is a rare eye disease that leads to severe visual impairment. LHON is a maternally inherited optic neuropathy caused by mitochondrial DNA mutations (3–6) and it affects approximately 1 in 30–000 to 1 in 50–000 individuals (7–9). The penetrance is incomplete and differs between genders. Conversion from carrier to affected typically occurs between 15 and 35 years of age and is more common among males (10, 11). The subacute visual loss often begins unilaterally and becomes bilateral within a year, often progressing to severe visual impairment with central scotomas. Spontaneous recovery may occur to some extent, but the long-term outcome is often poor (12).

The incomplete penetrance of LHON is suggested to be due to genetic and environmental factors (13–15). The risk of disease onset was initially estimated to be as high as 50% for male carriers compared to 10% for female carriers. However, recent studies indicate a much lower risk (16–18). Modifiable factors that increase the risk include smoking and excessive alcohol consumption (19, 20). Epidemiological studies show that smoking increases the risk for asymptomatic carriers developing symptoms, and excessive alcohol consumption is also associated with increased risk (15, 21). It is therefore important to investigate awareness of these risk factors among the asymptomatic carriers.

Health-related quality of life (HRQoL) is a concept of multiple dimensions referring to “the subjective evaluation of the impact of the health status in domains related to physical, mental, emotional, and social functioning” (22). A previous study recommended HRQoL to be investigated also in rare diseases and demonstrated successful examples of the use of patient-reported HRQoL instruments in this field (22), while another study on HRQoL in diverse rare diseases, reported poorer scores (23). Onset of visual loss in LHON carriers has been demonstrated to affect both generic health related and vision-specific QoL (19, 24, 25). Even in normally sighted carriers, the HRQoL was negatively impacted (19). QoL is also shown to be impacted in subjects with visual impairment due to common eye diseases (26–28). A report in low vision cohorts found greater difficulties with self-care, daily activities, pain, anxiety and depression, compared to a reference population, although the underlying cause of visual loss did not significantly influence QoL (26).

To our knowledge, there are no previous studies on QoL, alcohol and tobacco dependency on the Swedish LHON population. This study investigates QoL in both LHON affected and carrier cohort, with a generic health related QOL questionnaire. In Sweden, the use of smokeless tobacco (snus) is common, and its impact on LHON is also important to investigate given the roles of smoking and nicotine in degenerative eye disease. This study also investigates tobacco and alcohol consumption in LHON cohort. For comparison, we included two age and sex matched reference groups: one group without any known ocular diseases, and another group affected with ocular diseases other than LHON.

## 2 Materials and methods

### 2.1 Recruitment of study population

#### 2.1.1 LHON affected and asymptomatic carriers

All individuals admitted in the Swedish national research registry (the Swedish LHON registry -Svenska LHON-registret) was invited to participate in this cross-sectional case-control study. Data was collected between May and December of 2023. In total 75 LHON affected and 76 carriers who have given consent to be contacted were approached through email. The carriers had no history of any symptoms related to LHON disease conversion. The email invitation included a link to a secure online data collection platform. In total 32 LHON patients (43%) and 32 carriers (42%) filled in the questionnaires on the data collection platform. In the LHON group the majority (about 90%) were in the chronic stage of the disease.

#### 2.1.2 Reference groups

For reference groups, data were collected from the general population through recruitment announcement posted on the Karolinska Institutet’s website for study participants. Individuals with visual impairment due to other causes were also targeted through Karolinska Institutet’s website announcement and through different patient organizations. Self-reported data on visual status and diagnosis were recorded that may differ from objectively measured visual acuity, The data collection was done on the same digital platform. From the reference groups questionnaire responses, data was stratified with controlled randomization to obtain age and sex matched controls to the LHON affected and carriers with a target of 2:1 ratio for healthy controls (HC) and close to 1:1 ratio for other eye diseases (OED). After the stratification process, we reached the targeted age and sex matched reference group for HC, and about two-third of the targeted age and sex matched references for the OED reference group and proceeded with further analysis with this data.

### 2.2 Data collection

All questionnaires were initially compared for paper and digital version to assess the usability for electronic use. The majority of the questionnaires used in this study, or comparable versions of questionnaires, have previously been used to examine individuals with LHON and asymptomatic carriers, as well as within other population experience vision loss (2, 19, 29, 30). All questions were administered in Swedish.

#### 2.2.1 Demographics and various health parameters

The survey included questions on demographics and social characteristics (employment and family), other known health conditions/current diagnoses, and geographical location. LHON affected and asymptomatic carriers were also asked about their awareness of smoking as a risk factor for disease conversion, whether they had family/relatives affected by LHON, and if awareness of being affected/carrier had influenced their smoking and drinking habits, choice of home, choice of work, and family planning. Information on established risk factors, including tobacco smoking and excessive alcohol consumption, was provided in written form to patients and carriers at study inclusion; however, due to the absence of a standardized national protocol, the timing, format, and content of any information provided prior to inclusion were not systematically recorded.

#### 2.2.2 The RAND-36 measure of HRQoL

RAND-36 was used to assess HRQoL by investigating functional and emotional well-being. RAND-36 has been translated to Swedish and validated (31). We calculated the mental composite summary (MCS) and physical composite summary (PCS) (32, 33). We used an unweighted PCS and MCS score, with a higher score indicating a more preferrable health state. 97% of the affected subjects and 94% of the carriers completed the RAND 36 questionnaire.

Fagerström Test for Nicotine Dependence (FTND) for smoking and smokeless tobacco (snus) dependency.

FTND for smoking and snus dependency was used to investigate self-reported smoking and snus habits and to measure nicotine dependence. The proportion of smokers and snus users, for both affected and LHON carriers was compared to the prevalence of reference groups. For dependency the total score was calculated according to the FTND manual and categorized as very high, high, moderate, low or very low dependency. In both LHON groups, 94% completed the FTND questionnaire.

#### 2.2.3 Alcohol use disorder identification test

AUDIT was used to identify alcohol dependence and excessive alcohol consumption. The scores were calculated according to the AUDIT manual and categorized as low-risk consumption, hazardous or harmful consumption, likelihood of alcohol dependence or moderate-severe alcohol use disorder. 75% of the affected subjects and 84% of the carriers completed the AUDIT questionnaire.

### 2.3 Statistical analysis

The statistical analysis was performed with JASP (0.16.3). Descriptive statistics are presented as mean ± standard deviation and median with interquartile range. Normal distribution of the data was tested with Shapiro-Wilk test. Continuous data was compared with independent samples t-test (Student or Mann-Whitney) and ANOVA (student or Kruskal-Wallis) with a Tukey or Dunn’s *post hoc* test. Nominal data were compared by Chi-square tests. Statical analyses were stratified by sex and treatment status. A p-value of less than 0.05 was considered statistically significant.

### 2.4 Ethical permission

The study protocol adhered to the tenets of the Declaration of Helsinki and was approved by the Swedish Ethical Review Authority (Dnr 2021-06402–01 and Dnr 2023-01105-02). A consent form and information on the study were included in the secure link to the data collection platform.

## 3 Results

### 3.1 Demographics

In total, answers from 233 individuals were included in this study (32 LHON affected, 32 carriers, 128 healthy controls, and 41 individuals with other ocular diseases than LHON). Age ranged between 18–70 years and 56% were males. **Table 1** presents data on subject demographics including primary LHON mutations of mitochondrial DNA for the LHON affected and carriers, affected relatives, awareness of mutation and its influence on occupation status, housing and family planning.

**Table 1:** Demographic characteristics of the participants.

Variables	LHON affected	LHON carriers	HC reference for affected	HC reference for carriers	OED reference for affected	OED reference for carriers
Subjects, n	32	32	64	64	20	21
Males, n (%)	20 (62.5)	10 (31.3)	33 (51.6)	18 (28.1)	8 (40.0)	5 (23.8)
Age, mean ± SD (y) Median [range]	49.1 ± 19.7 43.5 [18-87]	48.2 ± 17.7 48.5 [20-77]	47.9 ± 19.4 43.5 [18-87]	47.9 ± 18.0 48.5 [20-77]	58.3 ± 17.8 64.0 [21-83]	52.5 ± 16.3 50.0 [27-76]
Female Age, mean ± SD Median [range]	54.5 ± 13.1 57.5 [34-73]	49.1 ± 18.5 51.5 [20-77]	57.6 ± 17.1 59.0 [18-87]	48.9 ± 17.9 51.0 [26-75]	63.8 ± 14.8 66.0 [38-83]	57.9 ± 14.4 62.5 [28-76]
Male Age, mean ± SD Median [range]	48.2 ± 22.1 41.0 [18-87]	46.1 ± 16.5 41.5 [26-75]	38.8 ± 20.0 35.0 [18-71]	45.4 ± 18.5 36.0 [26-76]	50.0 ± 19.6 52.5 [21-75]	35.4 ± 8.5 34.0 [27-49]
Visual Acuity,						
LogMAR, mean ± SD	1.4 ± 0.6					
I experience my visual acuity as… (%)						
Better than normal		9.4	14.1	7.8	0	0
Normal		37.5	40.6	45.3	0	0
Little less than normal		34.4	45.3	46.9	0	0
Less than normal		18.8	0	0	65.0	52.4
Much worse than normal		0	0	0	35.0	47.6
Other eye disease, diagnosis, (%)	N/A	N/A	N/A	N/A		
AMD/Retinal pathologies					19.8	9.5
Aniridia					20	9.5
Glaucoma					20	23.8
Cataract					15	9.5
Corneal diseases					0	19
Others *					25.2	28.7
Occupations, %						
Employed	31.3	62.5	50.0	57.8	40.0	42.9
Student	15.6	3.1	15.6	9.4	5.0	4.8
Home with kids	0	3.1	0	1.6	5.0	0
Un-employed	6.3	3.1	3.1	1.6	5.0	9.5
Sick-leave/early retirement	21.9	0	4.7	4.7	10.0	9.5
Retired	25.0	28.1	25.0	25.0	35.0	33.3
Don’t know	0	0	0	0	0	0
Missing	0	0	0	0	0	0
Relatives affected, %			N/A	N/A	N/A	N/A
Siblings	25.0	53.1				
Parents	15.6	9.4				
Children	9.4	25.0				
Cousins	12.5	28.1				
No close relatives affected	37.5	3.1				
LHON awareness			N/A	N/A	N/A	N/A
How long have you known about carrying mutation?, %						
Less than 1 year	3.1	12.5				
Between 1 and 2 years	3.1	9.4				
Between 2 and 5 years	12.5	15.6				
Between 5 and 10 years	15.6	18.8				
More than 10 years	62.5	40.6				
Missing	3.1	3.1				
LHON onset		N/A	N/A	N/A	N/A	N/A
For how long have you had LHON?, %						
Less than 1 year	6.2					
Between 1 and 2 years	3.1					
Between 2 and 5 years	12.5					
Between 5 and 10 years	15.6					
More than 10 years	56.3					
Missing	6.3					
Have LHON conversion/awareness of being a carrier influenced (partly/definitely) you regarding			N/A	N/A	N/A	N/A
Where you live	21.9	6.3				
Housing	21.9	3.1				
Family planning	15.6	25.0				
Choice of occupation	53.1	6.3				
m.11778G>A, n (%)	22 (68.8)	22 (68.8)	N/A	N/A	N/A	N/A
m.3460G>A, n (%)	2 (6.3)	1 (3.1)	N/A	N/A	N/A	N/A
m.14484T>C, n (%)	1 (3.1)	0 (0)	N/A	N/A	N/A	N/A
Treated with idebenone, n (%)	13 (40.6)	N/A	N/A	N/A	N/A	N/A

LHON, Leber hereditary optic neuropathy; HC, Healthy controls; OED, other eye diseases; N/A, not applicable; SD, standard deviation; y, years; * Others includes Diabetes retinopathy, Retinal detachment, Iritis, Congenital eye diseases, Optic nerve infarction.

Thirty-two affected LHON subjects participated, 20 (62.5%) were male (male:female ratio 1.7:1). Mean age at data collection was 49.1 ± 19.7 years (range 18 - 87). Of the 32 LHON subjects, two subjects (6.3%) were affected with LHON less than a year ago, classifying these two as in the sub-acute phase of the disease. 23 subjects (71.9%) were affected at least five years ago, and two subjects’ disease duration were missing. Of the affected LHON subjects, 37.5% had no other relatives affected by the disease. Of the 32 affected, 13 (40.6%) had received idebenone, and 2 (6.3%) had received q10.

Of 32 carriers, 10 (31.3%) were male (male:female ratio 0.5:1). Mean age at data collection was 48.2 ± 17.7 years (range 20 - 77) and the majority (59.3%) had known about carrying a LHON mutation for at least five years. Seven subjects (21.9%) had known about carrying a LHON mutation for less than two years, and one subject had no data on duration.

More LHON affected individuals were influenced on their occupation status, housing, and family planning by their diagnosis (53.1%, 21.9% and 25.0% respectively) compared to LHON carriers due to their awareness of being a carrier (6.3%, 6.3%, and 15.6% respectively). However, only occupation status was significantly different (p<0.001, X^2^ = 4.425, chi-square). A higher prevalence of sick leave, early retirement, and unemployment was seen among LHON affected.

### 3.2 HRQoL

**Table 2** shows the RAND-36 scores for the eight subscales, MSC, PSC and the perceived change in health for a 12-month period. While comparing the affected subjects with their age and sex-matched HC and OED, no significant differences were observed (p>0.05, ANOVA) for any of the RAND-36 domains. Carriers showed no significant differences with their age and sex matched HC, but their OED reference group showed lower scores on several subscales: PCS (p = 0.020), General Health (p = 0.033), and Role Limitations due to Physical Health (p = 0.026).

**Table 2:** RAND-36 scores.

Domains Mean ± SD Median [interquartile range 25-75]	LHON affected	LHONcarriers	HC reference for affected	HC reference for carriers	OED reference for affected	OED reference for carriers
Physical functioning	88.1 ± 21.7 95.0 [90.0-100]	88.2 ± 16.2 95.0 [90.0-100.0]	87.5 ± 16.0 95.0 [80.0-100.0]	91.5 ± 12.1 95.0 [85.0-100.0]	84.2 ± 19.5 95.0 [73.8-96.3]	84.5 ± 14.9 85.0 [75.0-95.0]
Role limitations due to physical health	77.5 ± 33.1 100.0 [56.3-100]	82.5 ± 30.9 100.0 [75.0-100]	69.9 ± 40.1 100.0 [25.0-100.0]	86.7 ± 28.5 100.0 [100.0-100.0]	68.8 ± 32.2 75.0 [50.0-100.0]	59.5 ± 42.2 75.0 [25.0-100.0]
Role limitations due to emotional problems	67.8 ± 37.6 83.3 [33.3-100.0]	63.3 ± 41.3 83.3 [33.3-100]	65.6 ± 38.5 66.7 [33.3-100.0]	71.9 ± 37.2 100.0 [33.3-100.0]	76.7 ± 30.8 100.0 [66.7-100.0]	63.3 ± 43.1 83.3 825.0-100.0]
Energy/Fatigue	62.8 ± 16.1 66.7 [55.0-75.0]	55.8 ± 19.2 52.5 [40.0-73.8]	55.6 ± 20.5 55.0 [43.8-70.0]	60.7 ± 21.5 65.0 [48.8-80.0]	62.5 ± 17.8 62.5 [48.8-76.3]	54.8 ± 28.3 60.0 [40.0-75.0]
Emotional Well-being	70.7 ± 20.7 72.0 [64.0-88.0]	68.9 ± 19.1 72.0 [53.0-84.0]	71.6 ± 17.0 76.0 [60.0-84.0]	74.9 ± 17.5 78.0 [64.0-88.0]	75.6 ± 14.4 76.0 [67.0-88.0]	64.4 ± 27.2 68.0 [52.0-84.0]
Social functioning	80.4 ± 25.8 100.0 [65.6-100.0]	76.7 ± 21.0 75.0 [62.5-100.0]	78.3 ± 22.4 87.5 [62.5-100.0]	83.4 ± 21.9 87.5 [71.9-100.0]	78.1 ± 25.3 87.5 [62.5-100.0]	66.7 ± 32.2 75.0 [37.5-100.0]
Pain	77.1 ± 25.1 80.0 [58.1-100.0]	77.9 ± 22.1 82.5 [67.5-100.0]	76.3 ± 20.7 80.0 [65.6-90.0]	80.6 ± 17.9 88.8 [67.5-90.0]	75.4 ± 24.7 80.0 [65.0-90.0]	68.2 ± 23.0 67.5 [45.0-90.0]
General health	68.3 ± 18.8 75.0 [55.0-80.0]	72.0 ± 20.1 75.0 [60.0-90.0]	64.7 ± 20.1 65.0 [50.0-80.0]	70.3 ± 22.0 72.5 [55.0-90.0]	66.0 ± 21.2 65.0 [53.8-86.3]	56.0 ± 24.6 60.0 [35.0-75.0]
Health change	53.9 ± 25.5 50.0 [50.0-75.0]	43.8 ± 16.8 50.0 [50.0-50.0]	56.6 ± 21.0 50.0 [50.0-75.0]	53.1 ± 17.5 50.0 [50.0-75.0]	48.8 ± 15.1 50.0 [50.0-50.0]	50.0 ± 23.7 50.0 [25.0-75.0]
Mental composite summary	70.0 ± 19.7 71.8 [60.1-85.9]	66.2 ± 20.5 65.7 [45.0-86.8]	67.8 ± 20.8 73.0 [51.1-83.7]	72.7 ± 21.1 80.5 [59.1-89.8]	73.2 ± 17.6 74.6 [65.4-88.1]	60.9 ± 29.5 66.4 [36.9-86.9]
Physical composite summary	77.9 ± 81.6 81.6 [70.0-90.9]	80.1 ± 17.5 87.5 [68.6-91.7]	74.6 ± 19.6 81.3 [61.6-88.1]	82.3 ± 15.6 86.6 [75.9-92.5]	73.6 ± 20.7 76.6 [67.9-88.4]	67.1 ± 22.6 65.0 [45.0-88.8]

LHON, Leber hereditary optic neuropathy; HC, Healthy controls; OED, other eye diseases; SD, standard deviation; *Affected subjects compared with HC and OED groups (all p > 0.05, ANOVA). Carriers showed no differences compared with HC (all p > 0.05), but had higher Physical Component Summary, General Health, and Role Limitations due to Physical Health scores than their OED reference group (p = 0.020, p = 0.033, and p = 0.026, respectively; ANOVA).

**Figure 1:**
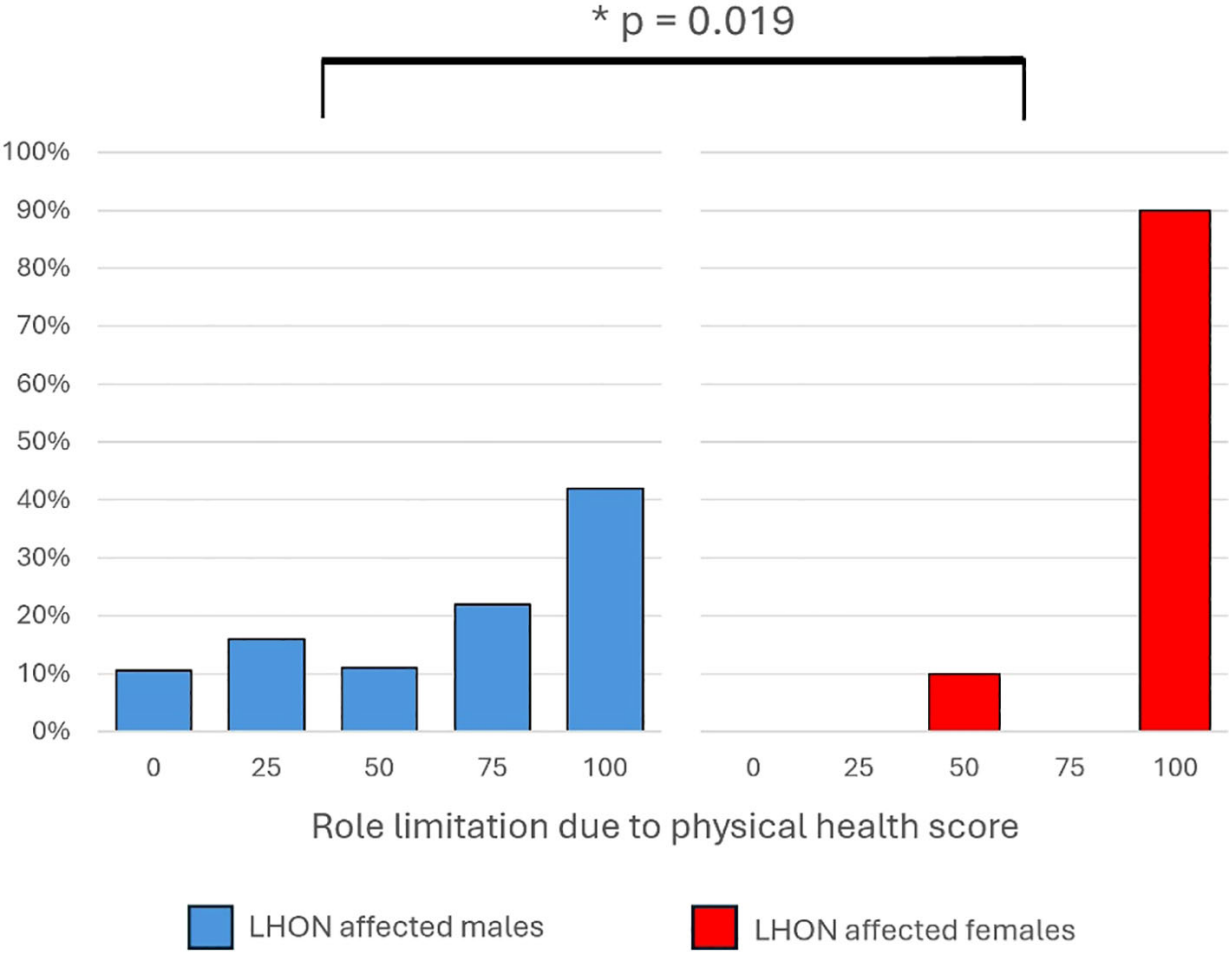
Domain score for role limitation due to physical health. Distribution in percentage for Leber hereditary optic neuropathy (LHON) affected males and females *p = 0.019.

No significant differences were found between LHON affected and carriers even after stratifying for sex (p>0.05, independent samples t-test; RAND-36 scores for LHON affected and carriers stratified by gender see **Supplementary Table 1**). However, the affected males had a lower score on role limitations due to physical health than affected females (67.1 ± 36.4 compared to 95.0 ± 15.8) (p = 0.019) (**Figure 1**). The other subscales show no statistical differences, even after stratifying for sex, and there were also no significant differences in HRQoL scores between treated and untreated LHON affected subjects ((p>0.05). Carriers showed the lowest score and a negative change in the subscale health change, but it was not statistically significant (p = 0.078). The only other group with a negative change is the OED reference group matched to the LHON affected group, but the differences are not statistically significant.

### 3.3 Smoking and snus use

The self-reported smoking and snus-use are presented in **Table 3**. The prevalence of smokers was higher in the LHON affected group (20%) than in the carrier group (10%). In both groups 78.1% had knowledge of smoking being a risk factor for LHON conversion. Both prevalence and awareness were not significantly different between the groups (p>0.05). For male participants 20% of the affected and 11% of carriers were current smokers. For females the prevalence of current smokers was 18% and 9.5% for the affected and carriers respectively. In all the current smokers in both groups, the smoking habit had started before awareness of carrying LHON mutation. The reference groups had lower prevalence of current smokers, which ranged from 1.6% to 5.3%. Compared to their reference groups the affected individuals displayed significantly higher prevalence of current smokers (p = 0.045 X^2^ = 6.181). However, the carriers did not significantly differ from their reference groups (p = 0.175 X^2^ = 3.489). A higher prevalence of non-smokers (never smoked) was found in the LHON carrier group compared to the affected group (59.4% vs 34.4%). Of the affected subjects, 6.3% stopped smoking after learning about carrying a LHON mutation, and 6.3% stopped after disease conversion. Of the carriers 3.1% stopped smoking after learning about carrying a mutation. To become aware of the LHON mutation has influenced the habit of smoking, in 43.7% of the affected, and 37.5% of the carriers.

**Table 3:** Smoking and smokeless tobacco.

Variables	LHON affected	LHON carriers	HC reference for affected	HC reference for carriers	OED reference for affected	OED reference for carriers
Current smokers, %	20.0	10.0	4.8	1.6	5.3	5.0
Males, %	22.2	11.1	3.1	5.6	0	25.0
Females, %	18.2	9.5	6.5	0	8.3	0
EX-smokers, %			N/A	N/A	N/A	N/A
Quit smoking…						
after awareness of mutation	6.3	3.1				
after LHON disease conversion	6.3	N/A				
because of other medical event	0	3.1				
other reason	18.8	25.0				
Missing	11.0	9.4				
Never smokers, %	34.4	59.4	N/A	N/A	N/A	N/A
Smokers, %			N/A	N/A	N/A	N/A
Started smoking…						
prior to awareness of mutation	100.0	100.0				
after awareness of mutation	0	0				
after LHON disease conversion	0	0				
Has LHON conversion/awareness of being a carrier influenced you regarding smoking?, %			N/A	N/A	N/A	N/A
No	56.3	62.5				
Partly	12.5	18.8				
Definitely	18.8	15.6				
Missing	12.5	3.1				
Smoking is a known risk factor for LHON conversion, %			N/A	N/A	N/A	N/A
Yes, I know that smoking is a risk factor.	78.1	78.1				
No, I didn’t know smoking is a risk factor	12.5	21.9				
Missing	9.4	0				
Classification on dependence based on FTND, for current smokers, %						
Very High	0	0	0	0	0	0
High	0	0	33	0	100	0
Moderate	0	0	0	0	0	0
Low	0	33	0	0	0	100
Very low	100	67	67	100	0	0
Snus-users, %	33.3	3.8	15.3	13.0	0	6.3
Classification on dependence based on FTND, for current snus-users, %					NA	
Very High	12.5	100	33.3	28.6		0
High	37.5	0	33.3	14.3		100
Moderate	12.5	0	22.2	14.3		0
Low	37.5	0	0	14.3		0
Very low	0	0	11.1	14.3		0
Missing	0	0	0	14.3		0

LHON, Leber hereditary optic neuropathy; HC, Healthy controls; OED, other eye diseases; FTND, Fagerström Test for Nicotine Dependence.

Prevalence of snus-users was higher for the affected (33.3%) compared to the carrier (3.8%) (p = 0.007 X^2^ = 7.352). For the current snus-users among the affected the classification on dependence ranged from low to very high. The LHON affected group had a significantly higher prevalence of snus-users, whereas the age and gender matched OED reference group had 0% and the HC reference group had 15.3% (p = 0.017, X^2^ = 8.147).

### 3.4 Alcohol consumption

Data on alcohol consumption is presented in **Table 4**. Among LHON affected and the carriers, more than 50% answered that being affected or knowing about the mutation, had not influenced their stand on alcohol. Among the affected and carriers, 28% and 44% respectively answered that becoming affected or being aware of carrying the mutation had partly or definitely influenced their stand toward alcohol. 25% of the affected subjects had a hazardous or harmful alcohol risk classification. This is higher than the carriers (7.4%) and the reference groups (9.6 to 17.2%). However, the difference is not statistically significant (p=0.745).

**Table 4:** Alcohol related data.

Variables	LHON affected	LHONcarriers	HC reference for affected	HC reference for carriers	OED reference for affected	OED reference for carriers
Has LHON conversion/awareness of being a carrier influenced you regarding alcohol?, %			N/A	N/A	N/A	N/A
No	59.4	53.1				
Partly	21.8	25.0				
Definitely	6.3	18.7				
Missing	12.5	3.1				
AUDIT score	3.3 ± 3.7	2.6 ± 3.1	4.1 ± 3.9	4.2 ± 3.7	2.7 ± 2.2	2.6 ± 3.6
AUDIT risk classification, %						
Abstainer	12.5	14.8	9.4	9.4	5	19
Low	62.5	77.8	76.6	73.4	85	71.4
Hazardous or harmful	25.0	7.4	12.5	14.1	10	4.8
Alcohol dependence	0	0	1.6	3.1	0	4.8

LHON, Leber hereditary optic neuropathy; HC, Healthy controls; OED, other eye diseases; AUDIT, the Alcohol Use Disorder Identification Test.

## 4 Discussion

The main aim of current study was to investigate HRQoL and alcohol and tobacco dependence in individuals affected with visual loss due to LHON and carriers in Sweden. To our knowledge this is the first study on HRQoL and tobacco and alcohol habits in the Swedish LHON cohort. The findings from this investigative study indicate that the LHON affected and carriers did not have any significant difference in HRQoL compared to the reference HC group. The reference HC group had similar HRQoL scores as previously published Swedish reference data hence can be considered representative (34). In the LHON affected group, males had a significantly lower score for role limitation due to physical health than females. A higher prevalence of smoking and snus use as well as alcohol consumption was seen among individuals affected with LHON compared to carriers and the reference groups.

The majority of the LHON affected individuals included in the current study experienced vision loss more than 10 years ago. Approximately two-thirds of these conversions occurred before the authorization of idebenone for LHON treatment in Europe (September 2015). Among those who experienced conversion within the last 10 years, the majority (10 out of 12) received idebenone. In our previous study, we found that the treated individuals had better visual acuity compared to untreated (35). In current study, we did a sub-analysis to evaluate if there are any differences in HRQoL between treated and untreated LHON affected subjects and found no significant differences (p>0.05).

When stratifying for sex, the LHON affected males showed lower values in role limitations due to physical health compared to the affected females. This domain score did not vary among affected and carriers. When comparing the LHON affected and carriers with respective OED reference groups (majorly comprised of age-related macular degeneration and glaucoma) we did not observe any large differences in HRQoL (**Table 2**). A previous report on low vision subjects suggested that the different causes of low vision provided similar HRQoL (26). The LHON affected subjects from the current cohort had a similar HRQoL median scores for general health and role limitations due to physical health but a lower score for role limitations due to emotional health and emotional well-being compared to a previous report that included patients with glaucoma and retinitis pigmentosa (29). It has also been reported that rare diseases in general might impact the QoL further (23) though we did not see this trend. In rare diseases the main challenge is the delay in obtaining a definite diagnosis as well as limited treatment options. All the subjects included in the Swedish LHON registry have received their diagnosis or are aware of being a carrier.

Though previous studies have reported that LHON subjects showed reduced QoL (24, 25, 36, 37), our results show similar values between the LHON cohort and healthy controls. This could be due to the differences in the questionnaire administered as our aim is to evaluate the generic aspects of HRQoL, and hence we used RAND 36 (free, public domain version of SF-36). Swamy et al. showed that the NEI-VFQ25 subscales and SF-36 subscales including the physical and mental composite scores showed a low correlation (38). Our affected subjects are mainly in the chronic stage of the disease and coping and acceptance could be better at this stage. It is also possible that the Swedish LHON cohort experiences a better HRQoL due to the societal factors such as disability friendly infrastructure, high accessibility and well-established healthcare system (39–41). It should also be noted that only 43% of affected individuals contacted filled in the questionnaires and this could have led to a recruitment bias.

It is crucial to better understand the factors affecting HRQoL among the carriers. Although only a small proportion may experience conversion to vision loss, the awareness of being at risk can cause mental stress, and among the women the knowledge of passing it on to their children could cause extra concerns. A previous study on a German cohort showed that female carriers had a much higher prevalence of mild to moderate depression compared to the general population (19). In the present study, MCS did not show any difference between the female and male carriers or the affected individuals. However, 25% of the carriers (33% males and 22% females) responded that their family planning was influenced by their awareness of being a carrier.

The affected subjects had a higher prevalence of smoking compared to the other groups, a result that possibly supports the suggested association between smoking and LHON conversion (19–21). The prevalence of smoking in our control groups is close to that of the reported prevalence of regular smokers (6%) in Sweden (42). Comparing the proportion of individuals that never smoked among the LHON affected and carriers, the prevalence was higher among the carriers, with about six-tenths that never smoked. In our cross-sectional study, 10% of the carriers are current smokers and about a third have stopped smoking. For the LHON affected individuals one-fifth are current smokers, and one-third report themselves as never-smoked. All current smokers in the LHON affected and carrier groups reported that they started smoking before they were aware of carrying the mutation. Only 6.3% of the LHON affected quit smoking after they were aware of carrying the mutation and another 6.3% quit after the conversion. Majority of subjects who have quit smoking reported that they did so due to other reasons. A previous study reported that about one-third of LHON patients continued with smoking even after they were aware of the mutation (19). A reason for this might be the lack of knowledge that smoking is a risk factor. In our LHON groups, 13.8% of the affected and 21.9% of the carriers were unaware of smoking being associated with a higher risk for disease conversion. Previous study (19) also reported a small improvement in visual acuity for 45% of the LHON affected patients that stopped smoking.

A quarter of all LHON affected are using snus (smokeless tobacco), and 62.5% of those fall under moderate to very high dependency based on FTND classification. This high dependency might be a result of the Swedish cultural stand on allowing and using snus. In Sweden, snus usage is more common among males, however a recent report shows that snus usage is increasing among females (43).The limited data on snus and LHON, restricts the possibility to analyze snus as a factor for disease development. To our knowledge this is the first report on baseline data on the prevalence of snus usage among the LHON population in Sweden.

A quarter of all LHON affected had a hazardous or harmful alcohol risk classification, which is much higher than the carriers. More than half of the LHON affected and carriers responded that either the conversion or the awareness of carrying the mutation did not influence their attitude toward alcohol consumption. Similar to the findings in the German LHON cohort (19), LHON affected males had a higher proportion of harmful or hazardous alcohol risk classification. One third of LHON affected males were in these classifications compared to one eighth of the females. Even among the carriers, males had a higher proportion of hazardous or harmful alcohol consumption compared to females (12.5% and 5.2% respectively). However, the male carriers had the highest proportion of abstainers with a quarter not drinking alcohol, compared to 13.3% for the affected males, and 12.5% and 10.5% for the affected and carrier females.

In Sweden there are no protocols for how to care for and monitor the carriers and there are no formal recommendations on information to be provided. The German study approach (19) with longitudinal visits indirectly offers a care-program on regular basis to both LHON affected and carriers. In Sweden, if such a system is implemented with regular clinical follow-ups it could be helpful for the carriers and bring awareness on the risk factors. However, regular follow-ups for the carriers might also introduce stress.

Our study has some limitations, as it is a cross-sectional design, and the subjects may have adjusted differently over time to the awareness of carrying the mutation or disease conversion. Only two LHON affected subjects were in the sub-acute stage of the disease at the time of data collection, and only about one-fifth of the carriers had learnt about being a carrier in the past two years. Therefore, the results cannot be extrapolated to map the HRQoL closer to the time of conversion or the emotional responses associated with learning about carrying the mutation. In addition, the data on alcohol consumption and tobacco dependence are self-reported, which may lead to underreporting. It would also be interesting to know the subjects’ habits prior to disease conversion, but this was not assessed in order to avoid recall bias. Hence, no interpretation can be done on how environmental modifiers could have contributed to a conversion event.

## 5 Conclusion

The LHON affected and carriers in the Swedish cohort, reported similar HRQoL compared to healthy controls. Among the LHON affected group, males had a significantly lower scores for role limitation due to physical health. LHON affected and carriers showed a higher prevalence of smoking. LHON affected individuals also showed higher snus usage, and harmful or hazardous alcohol consumption.

## Data Availability

The original contributions presented in the study are included in the article/**Supplementary Material**. Further inquiries can be directed to the corresponding author.
