# Supplementary material for: Health-related quality of life and tobacco and alcohol consumption in Leber hereditary optic neuropathy in Sweden
Source: Front Ophthalmol (Lausanne). 2026 Jun 15;6:1791334. doi: 10.3389/fopht.2026.1791334 (PMC13310744; doi:10.3389/fopht.2026.1791334)
Supplement: Supplementary file 1 [file Table1.docx]

**Table S1.** RAND-36 scores for LHON affected and carriers

| Domains  Mean±SD  Median [interquartile range 25-75] | LHON affected Males | LHON affected Females | LHON carrier Males | LHON carrier Females |
| --- | --- | --- | --- | --- |
| Physical functioning | 90.0 ± 17.8  95.0 [93.8-100.0] | 83.0 ± 29.1  92.5 [85.0-100.0] | 92.2 ± 16.2  100.0 [95.0-100.0] | 86.4 ± 16.2  90.0 [75.0-100.0] |
| Role limitations due to physical health | 67.1 ± 36.4  75.0 [37.5-100.0] | 95.0 ± 15.8  100.0 [100.0-100.0] | 86.1 ± 33.3  100.0 [100.0-100.0] | 81.0 ± 30.5  100.0 [50.0-100.0] |
| Role limitations due to emotional problems | 57.9 ± 39.8  66.7 [33.3-100.0] | 83.3 ± 28.3  100.0 [75.0-100.0] | 59.3 ± 43.4  66.7 [33.3-100.0] | 65.1 ± 41.5  100.0 [33.3-100.0] |
| Energy/Fatigue | 62.6 ± 18.3  70.0 [50.0-75.0] | 62.4 ± 12.3  60.0 [55.0-75.0] | 62.2 ± 20.9  75.0 [45.0-75.0] | 53.1 ± 18.2  50.0 [40.0-70.0] |
| Emotional Well-being | 67.4 ± 23.4  72.0 [50.0-84.0] | 75.8 ± 13.5  72.0 [70.0-88.0] | 69.8 ± 22.5  80.0 [56.0-84.0] | 68.6 ± 18.1  72.0 [52.0-84.0] |
| Social functioning | 75.0 ± 26.4  75.0 [56.3-100.0] | 88.8 ± 23.9  100.0 [90.6-100.0] | 77.8 ± 27.1  87.5 [62.5-100.0] | 76.2 ± 18.5  75.0 [62.5-87.5] |
| Pain | 71.4 ± 27.0  77.5 [57.5-95.0] | 85.5 ± 19.2  95.0 [67.5-100.0] | 83.9 ± 26.2  100.0 [87.5-100.0] | 75.4 ± 20.3  70.0 [67.5-90.0] |
| General health | 68.3 ± 19.4  75.0 [48.8-80.0] | 66.4 ± 18.1  69.4 [60.0-73.8] | 76.7 ± 17.3  80.0 [65.0-85.0] | 70.0 ± 21.3  75.0 [60.0-90.0] |
| Health change | 55.0 ± 26.4  50.0 [43.8-75.0] | 57.5 ± 20.6  50.0 [50.0-68.8] | 45.0 ± 19.7  50.0 [50.0-50.0] | 43.2 ± 15.8  50.0 [50.0-50.0] |
| Mental composite summary | 65.7 ± 21.7  68.4 [52.6-84.1] | 76.8 ± 12.3  81.8 [65.5-84.2] | 67.3 ± 25.6  81.4 [43.8-87.9] | 65.7 ± 18.7  63.7 [53.8-84.1] |
| Physical composite summary | 74.5 ± 18.7  81.3 [65.9-86.6] | 82.5 ± 14.0  90.0 [74.9-92.5] | 84.7 ± 17.7  90.0 [87.5-95.0] | 78.2 ± 17.4  83.8 [60.6-89.4] |

LHON, Leber hereditary optic neuropathy
